# A community-centric multi-disciplinary education program with the 8-section brocade Tai Chi therapy for patients with osteoarthritis of the knee – a pilot study

**DOI:** 10.1186/s12906-021-03480-2

**Published:** 2021-12-14

**Authors:** Kevin Ki-Wai Ho, Gerald Pong, Queena Wai-Chin Poon, Jojo Yan-Yan Kwok, Wai-Wang Chau, Michael Tim-Yun Ong

**Affiliations:** 1 Department of Orthopaedics and Traumatology, Chinese University of Hong Kong, Prince of Wales Hospital, Shatin, Hong Kong SAR, China; 2 School of Nursing, LKS Faculty of Medicine, The University of Hong Kong, Hong Kong SAR, China

**Keywords:** Tai Chi, Knee, Osteoarthritis, Multi-disciplinary education program

## Abstract

**Background:**

Osteoarthritis (OA) of the knee is one of the most common chronic degenerative joint diseases, and a multi-disciplinary approach to educating patients with OA knee are effective in symptoms management. Tai Chi exercise is a novel approach to relieving knee OA symptoms. Combining both educational program and Tai Chi has not yet been explored.

**Methods:**

Multi-disciplinary education program included a total of 4-week 2-h weekly talks delivered by different health professionals with live demonstrations. This was then followed by a 1-h Tai Chi class (Baduanjin). Results from IPAQ (Physical activity level), WOMAC (evaluate knee OA conditions), and SF-36v2 (quality of life) were collected at the first class of education program, 3 and 6 months after the end of Tai Chi class. CSQ-8 (program effectiveness) was administered on the last day of Tai Chi class.

**Results:**

One hundred and twelve patients joined the program. The overall attendance was over 90% with close-to-zero dropout rate. Satisfaction scored high in 85% of patients. WOMAC pain scores (*p* = 0.04) and SF-36v2 emotional role (*p* = 0.02) were statistically decreasing (improving) at 6 months after the program. SF-36v2 physical role and mental health tended to improve with time.

**Conclusions:**

Combining both multidisciplinary education program program and Tai Chi exercise for knee OA patients was proven feasible. This program received high satisfaction, high attendance and very low dropout rates without any adverse event. Patients’ pain and emotion were significantly improved. A large-scale randomized trial introducing a control group is recommended.

**Trial registration:**

Registry: ClinicalTrials.gov

Registration number: NCT04204213

Date of registration: 18/12/2019 (Retrospectively registered)

## Background

Osteoarthritis (OA) of the knee is one of the most common chronic degenerative joint diseases, primarily affecting the ageing population, limiting joint movement and causing disability because of pain and stiffness. The prevalence of radiologic knee OA increased in proportion to age, reaching 64.1% for those who were aged 60 and over, and had a higher prevalence in females than males [1]. Patients suffering from knee OA often encounter stiffness, swelling and pain. These symptoms eventually lead to overly sedentary lifestyles, weight gain and a decrease in physical activities [2].

Numerous studies investigated the efficacies of non-operative knee OA management aiming to slow down the deterioration of knee OA, which involved lifestyle and behavioural modifications, exercise and physiotherapy, weight reduction, injury avoidance, and pharmacological treatment [3–7]. Meditative exercises, such as Tai Chi, became trendy amongst the older population over the past few decades. Tai Chi is a complex multi-component mind-body exercise combining deep diaphragmatic breathing and relaxation with slow, gentle and graceful movements. The benefits of Tai Chi on bone health and fall prevention, such as enhancing senior patients’ bone mineral density and fragility fractures, have been well documented [8–10].

Studies elaborating the effects of Tai Chi on Knee OA have been built upon the potency of adverse symptoms mitigation with findings on WOMAC pain and physical function improvements [11, 12]. The performance of Tai Chi may influence the many concepts of beliefs that enhance the social and mental well-being of different aspects of qualities of life, and also mitigate against depression symptoms over time [11–15].

Knowledge empowerment through multi-disciplinary education approach has been evolving in recent years. Educating patients about their illnesses, with a focus on prevention and self-management, should be the foundation and basis of their treatment programmes [16]. This is particularly crucial for patients who are suffering from chronic illnesses, such as osteoarthritis of the knee [17], because effective long-term management primarily depends on the patient’s willingness to co-operate and their potential to adhere with treatments or management, such as physical exercise [18]. In addition, empowerment of patient knowledge is also listed in the European League against Rheumatism (EULAR) recommendation guidelines as being one of the most effective evidence-based management methods of knee OA [19]. Given the positive outcomes documented separately on patient education and Tai Chi exercises by previous studies, combining customised Tai Chi exercise specifically for knee osteoarthritis with multi-disciplinary education purposes could be a “buy-time” strategy trying to slow down the joint deterioration progression when the patients are waiting for surgery.

We developed a multi-disciplinary education program in conjunction with Tai Chi classes. The multi-disciplinary approach on educating and easing the difficulties resulted from OA knee is not a new idea, neither is the beneficial effect of Tai Chi on OA patients. The beneficial effects of Tai Chi (intervention) on those with advanced stages of knee OA see much potential despite the effect has yet to be alluded. Combining both education program (comprising different professional teams) and Tai Chi exercise has not been reported elsewhere. This study was a pilot study of the multi-disciplinary education and Tai Chi program of a large scale randomized controlled trial (RCT). The main objective of this study is to introduce and describe the multi-disciplinary education program and Tai Chi exercise in details and how they were carried out in the community. Outcomes of OA knee patients after this pilot study were briefly discussed afterwards.

## Methods

### Study participants

This study adopted a quasi-experimental design. Quasi-experimental design was adopted because this was a pilot study followed an empirical interventional study approach estimating the causal impact of an intervention (multi-disciplinary education program with the 8-section brocade Tai Chi) on OA knee patients without random assignment [20]. Patients were recruited at a specialised orthopaedic outpatient clinic from April 2015 to March 2016. The sample size followed the convenient sampling method [21]. Sample size was justified by the number of available OA knee patients meeting both inclusion and exclusion criteria from our pool of OA knee patients which had been described elsewhere [22]. Consecutive patients visiting the specialised orthopaedic outpatient clinic with symptomatic end-stage OA of the knee on queue for lower limb surgery were eligible to participate in this study. More than 150 patients confirming knee OA symptoms were primarily screened by orthopaedic specialists at their clinic follow-up visits. After the primary screening, the knee osteoarthritis was further confirmed by examining radiographs. The severity of OA was classified by Kellgren and Lawrence (KL) system [23] graded by an orthopaedic specialist. Inclusion criteria included: (1) diagnosed with symptomatic knee OA of KL grade 3 or 4, and had been on the waiting list for lower limb arthroplasty surgery, (2) aged 55 years or above, (3) were able to ambulate independently, and (4) were able to communicate in the local dialect. Exclusion criteria included: (1) previously having undergone total knee replacement surgery, (2) other non-OA forms of arthritis, (3) unable to complete all programme sessions and subsequence assessments. Diagnosis of OA knee was based on clinical and radiological finding based on the Therapeutic Criteria Committee of the American Rheumatism Association [24]. One hundred and twelve patients were enrolled in this study. All patients enrolled this study voluntarily and they did not receive any compensation. Ethical approval was obtained from the ethics review board of Joint Chinese University of Hong Kong – New Territories East Cluster Clinical Research Ethics Committee (Ref. No.: 2020.264). This study was registered at ClinicalTrials.gov (Study identifier: NCT04204213). Written informed consent was obtained from every participant. This study followed the Declaration of Helsinki.

### Multi-disciplinary education program and Tai Chi (Baduanjin)

Both activities lasted for a total of 2 months (8 weeks). The first month comprised the weekly 2-h multi-disciplinary education program followed by an hour of Tai Chi classes. Tai Chi classes continued weekly on the following month. Table 1 referred to the outline of the program.

**Table 1:** Outline and timeline of the Multi-disciplinary education program, Tai Chi classes, and different program evaluation tools

Events	Timeline						
	1st week (Baseline)	2nd week	3rd week	4th week	5th to 8th week	3 months after the 8-week program	6 months after the 8-week program
**Health talk**: Information on osteoarthritis and pain-management (Physician and nurse)	✓						
**Health talk and demonstration**: Lifestyle and physical activity (Physiotherapist)		✓					
**Health talk and demonstration**: Weight control and dieting (Dietitian)			✓				
**Health talk and demonstration**: Coping with activity restriction (Occupational therapist)				✓			
**Tai Chi class** (Weekly for 8 weeks)	✓	✓	✓	✓	✓		
**Health assessments** (WOMAC, SF-36v2, and IPAQ)	✓					✓	✓
**Program Satisfaction Evaluation** (CSQ-8)	✓			✓		✓	✓

Keys: *WOMAC* The Western Ontario and McMaster Universities Arthritis Index, *SF36v2* Short-Form 36 Version 2, *IPAQ* International Physical Activity Questionnaire, *CSQ-8* Client Satisfactory Questionnaire-8

#### Multi-disciplinary education program

The education programme consisted of health talks and live demonstrations. Each class lasted for 2 h, of which the first hour dedicated to health talks conducted by physicians, nurse, physiotherapists, dietitians or occupational therapists, and the second hour being live demonstrations supplementary to the health talks. For example, physiotherapists demonstrated exercises on muscle training after the talk, and a healthy cooking class followed after the weight control talk by dietitians.

#### Tai Chi (Baduanjin)

**Fig. 1:**
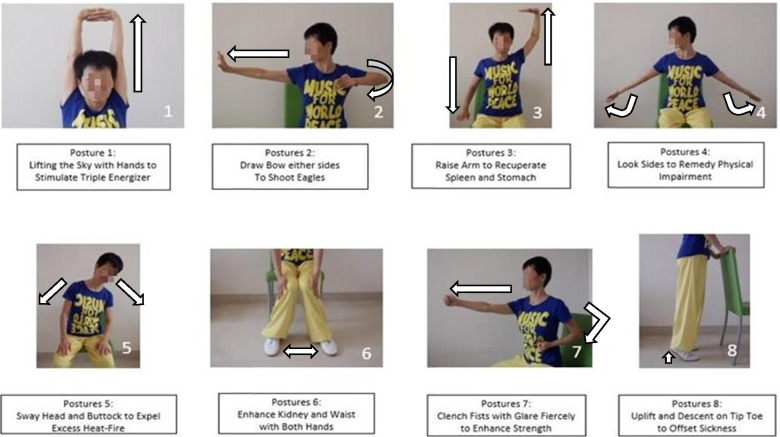
Illustrations of the 8 Section Brocade (Baduanjin) Tai Chi

Tai Chi exercise followed immediately after the education class. The Tai Chi exercise (Baduanjin) targeted patients with OA of the knee who were less mobile and have difficulties to stand. The Tai Chi master (a senior physiotherapist) demonstrated the knee OA-specific 8 section brocade Tai Chi exercise class (Fig. 1). The Tai Chi exercise lasted for 8 weeks (A weekly tutor-led 1-h Tai Chi session per week and lasted for 8 weeks). Every participant received a handout illustrating the movements at the end of the first tutor-led Tai Chi session (Fig. 1) as well as a video disc which the participants could review the Tai Chi movements by themselves. Outside the tutor-led Tai Chi session, participants were encouraged to practice on themselves daily for the rest of the week. Participants were asked to record their daily at-home practice on the appropriate spaces in the handout.

### Outcome measures

Health outcomes were measured and collected at baseline (the first class of the multi-disciplinary education program), 3 and 6 months after the 8-week Tai Chi classes (Table 1).

#### Primary outcomes

##### International physical activity questionnaire short form (IPAQ short form) [25]

IPAQ short form measures physical activity through physical activity metabolic equivalent of task (MET)-minutes per week based on 8 questions [25]. The results expressed in terms of low, moderate or high.

#### Secondary outcomes

##### Western Ontario and McMaster Universities Arthritis Index (WOMAC) [26]

WOMAC is a widely used standardised questionnaire to evaluate the condition of patients with OA of the knee and hip [26]. WOMAC composed of 24 questions and each question was on a 5-point Likert scale, of which “none” donated 0 point, “slight” denoted 1 point, “moderate” denoted 2 points, “severe” denoted 3 points and “extreme” donated 4 points. WOMAC Pain domain score was calculated by adding the scores from question 1 to 5. Adding the scores from question 6 and 7 created WOMAC Stiffness domain score, and WOMAC Function domain score was formed by summing up the scores from questions 8 to 24. WOMAC Total score was the sum of scores of all questions. WOMAC Pain domain score ranged from 0 (minimum) to 20 (maximum), Stiffness domain score ranged between 0 and 8, Function domain score ranged between 0 and 68, and Total domain score was between 0 (minimum) and 96 (maximum). The higher the WOMAC score means less level of disability and better health status (i.e. higher score means more pain).

##### Short Form-36 version 2 [27]

Short Form 36 Version 2 (SF-36v2) quantified the health outcomes in our OA knee patients focusing on their health conditions [28]. SF-36v2 measured health outcomes in terms of the following 8 domains: physical functioning, role-physical, bodily pain, general health, vitality, social functioning, role-emotional, and mental health. Physical Component Summary (PCS) and Mental Component Summary (MCS) scores were derived from the 8 domains. Domain scores ranged from zero to 100, of which a higher score indicates a better level of health (i.e. the higher the score the fewer disability). Details of the domain scores has been reported [29].

##### Client satisfaction questionnaire (CSQ-8) [30]

CSQ-8 assesses the effectiveness of the customised programme from the patient’s point of view [30]. Higher scores mean greater satisfaction.

### Data collection

IPAQ scores, WOMAC, and SF-36v2 scores were collected at baseline (the first class of the multi-disciplinary education program), 3 months and 6 months after the 8-week Tai Chi classes (i.e. 3 months and 6 months after the complete program). CSQ-8 was complete at the last Tai Chi class. Participants who were not able to return any questionnaire on-site were given envelopes to send them back later.

### Data analysis

Demographic statistics on age and sex were reported in terms of mean ± SD or frequencies where appropriate. Comparisons of categorized IPAQ outcomes were carried out using Chi-square test. Comparisons of WOMAC and SF-36v2 domain scores among the 3-time points were performed using ANOVA. *post-hoc* Bonferroni correction comparisons were carried out and presented using the log-rank test. Reporting using percentages was used for CSQ-8 results. A two-sided *p*-value ≤0.05 was considered statistically significant. All statistical analyses were carried out using IBM SPSS Version 26.0 (Armonk, NY: IBM Corp).

## Results

There were 36 males and 84 females with a mean age of 65.8 years old enrolled in this study. A complete data set (i.e. all three follow-ups) was available for 32 males and 80 females with a total of 112 patients (93.3%). Eight cases (6.6%) were lost to follow-up. Among the 112 patients, half received primary education, 39 (34.8%) educated to high school and the rest received college education or above. After the tutor-led Tai Chi session, over 90% of patients practiced Tai Chi on their own by reading the printed matters and video clips.

### IPAQ (Table 2)

Knee OA patients with high activity level remained the same percentage across the 3-time points. Percentages of low activity patients increased 3 months after the program and dropped at 6 months after despite the percentage was still higher than at baseline. No statistical difference was found.

**Table 2:** IPAQ scores in terms of low, moderate or high at baseline, 3 months and 6 months after the program

IPAQ	Time point			*P* value
	Baseline (N (Row %)) (Column %)	3 months after (N (Row %)) (Column %)	6 months after (N (Row %)) (Column %)	
Low	6 (18.8) (5.4)	15 (46.9) (13.4)	11 (34.4) (9.8)	0.373
Moderate	98 (35.0) (87.5)	89 (31.8) (79.5)	93 (33.2) (83.0)	
High	8 (33.3) (7.1)	8 (33.3) (7.1)	8 (33.3) (7.1)	

Reference on categorization of IPAQ scores: IPAQ official website http://www.ipaq.ki.se

*Chi-square test

### WOMAC and SF-36v2 (Table 3)

WOMAC pain, function, stiffness, and total domain scores were kept decreasing along the baseline, 3 months then 6 months after the program, of which statistical significance was found in the pain domain. Statistical significance was seen between baseline and 6 months (8.37 vs. 7.11, log-rank test *p* = 0.04). Similarly, similar significant changes were observed in SF-36v2 emotional role (53.79 vs. 43.82, log-rank test *p*-value = 0.02). Both physical role and mental health domains tended to reach statistical different (ANOVA *p*-values: physical role = 0.08; mental health = 0.08).

**Table 3:** WOMAC and SF-36v2 domain scores at baseline, 3 months and 6 months after the program

Domain scores	Baseline (Mean ± SD)	3 months after program (Mean ± SD)	6 months after program (Mean ± SD)	*P* value
WOMAC				
Pain	8.37 ± 3.72^a^	7.31 ± 3.88	7.11 ± 3.96^a^	0.03
Stiffness	3.31 ± 1.95	3.09 ± 2.02	3.24 ± 2.01	0.69
Function	29.38 ± 13.19	26.69 ± 14.23	26.17 ± 14.50	0.19
Total	41.05 ± 17.63	37.09 ± 19.36	36.77 ± 19.31	0.17
SF-36v2				
Physical function	37.14 ± 23.64	35.80 ± 25.50	38.30 ± 24.41	0.75
Physical role	44.08 ± 24.59	38.00 ± 25.14	37.39 ± 23.10	0.08
Bodily pain	54.91 ± 20.91	54.06 ± 23.90	56.45 ± 20.93	0.71
General health	41.92 ± 19.57	42.81 ± 18.04	42.77 ± 15.55	0.91
Vitality	45.09 ± 22.04	45.70 ± 21.39	45.48 ± 16.42	0.97
Social function	57.14 ± 27.20	55.47 ± 25.60	54.69 ± 23.62	0.76
Emotional role	53.79 ± 26.89^b^	49.93 ± 28.94	43.82 ± 27.33^b^	0.03
Mental health	59.64 ± 20.76	53.71 ± 21.81	55.58 ± 17.97	0.08
PCS	40.38 ± 22.14	39.79 ± 21.83	41.65 ± 18.78	0.80
MCS	51.08 ± 24.86	49.56 ± 22.85	49.20 ± 19.12	0.80

*WOMAC* The Western Ontario and McMaster Universities Arthritis Index, *SF-36v2* Short Form 36 Version 2, *PCS* Physical Component Summary, *MCS* Mental Component Summary, *SD* Standard deviation

^a,b^ANOVA with statistical difference after post hoc Bonferroni correction

### CSQ-8 (Table 4)

A total of 85.5% of patients felt highly/very likely the program help them deal with the problems (knee OA) and 73.3% felt the program was highly/very satisfactory in coping with their needs. A high percentage (77.7%) of patients would recommend the program. 86.6% of patients highly/very satisfied with the services (education program + Tai Chi exercise).

**Table 4:** Results from CSQ-8 questionnaire

Questions	Highly	Very	Fairly	Minimally	None		
Have the services you received helped you to deal more effectively with your problems	43.3%	42.2%	8.9%	1.1%	0%		
To what extent has our program met your needs?	38.9%	34.4%	23.3%	3.3%	0%		
Would you recommend the programme to others?	44.4%	33.3%	18.9%	3.3%	0%		
Questions	Highly satisfied	Very satisfied	Slightly satisfied	Fairly satisfied	Minimally satisfied	Fairly not satisfied	Highly Dissatisfied
How satisfied are you with the services you received?	42.2%	44.4%	6.7%	2.2%	0%	0%	0%

Our study recorded over 90% of overall class attendance rates. Patients who attended the first health talk and Tai Chi class and finished the first set of health assessments and program satisfaction evaluation completed the subsequent assessments at different time points. None reported adverse condition verbally after the Tai Chi class.

## Discussion

This is a pilot study to demonstrate the community-based, multi-disciplinary education program and a Tai Chi class on a small number of OA patients. Multi-disciplinary approach includes health concepts in the educational component that spanned across disciplines, and this pilot study tried out involving a physical activity component. The second component of this program was a 8-week Tai Chi class aiming for OA patients. No statistical difference in physical activity level, in terms of IPAQ results, was found after the program. Patients’ pain and emotion improved 6 months after the program. Quality of life in terms of physical ability and mental health improved despite both were not proved statistically. This pilot study received high percentages of patient satisfaction.

Multi-disciplinary approach on non-surgical management of OA knee was not a new concept. A systematic review on 4 European clinical trials were carried out in the UK, and concluded few studies employed multidisciplinary approaches in primary care suitable for OA across multiple joint sites [31]. Of the 4 selected studies, 2 were single-centre trials and 2 were multi-centre trials [32–35]. The patient education programme in the Swedish trial involved different health disciplines including physiotherapy, occupational therapy, orthopaedic specialist, nurse, and nutritionist [33]. This was a 5-week program with 3-h group sessions once a week [33]. The author concluded that patient education for OA patients was feasible in a primary health care setting. Self-perceived health and function was improved. The exercise program was recommended to include in the education program and looked for the effect on body function and self-efficacy [33]. The current study aimed to cohere the Swedish trial by introducing new elements on top of the multidisciplinary education programs. The differences between our study and Swedish study were the introduction of 1) demonstration sessions after every talk and 2) Tai Chi classes after the 4-week education program. We introduced demonstration sessions after the education sessions to gain interactive components as well as patients’ compliance and satisfaction with the overall program. The Tai Chi regimen forms the “exercise” component recommended by the Swedish team. Benefits coming through the program still exist after 6 months, especially in pain and emotion. Physical ability and mental health speculate to improve also. These results reflect the recommendations from the Swedish team that both talks and exercise program becomes a package when it comes to a community-based health promotion program. Results on improving body function and self-efficacy are proven effective. This kind of program design is also proven feasible aiming for a large scale RCT.

We adopt Baduanjin (The 8-section brocade Tai Chi) because it has been proven safe and effective on knee OA patients. Baduanjin proved to ease the knee OA symptoms from a randomized trial on 28 female knee OA (14 Baduanjin and 14 control) and concluded it is a safe and feasible exercise to reduce pain, stiffness and disability on knee OA patients [36]. Different randomized controlled trials on the effects of Baduanjin in knee OA patients further provided evidence that this specific Tai Chi exercise improves patients’ postural balance [37, 38], pain [37], stiffness [37], and function [38]. A systematic review and meta-analysis of 14 RCTs on the effectiveness of Traditional Chinese Exercise (Tai Chi, Baduanjin, Yijinjing, and Wuqinxi) for symptoms of Knee Osteoarthritis provided preliminarily positive influences for patients with symptoms of knee OA including pain, stiffness, and physical function [39]. In a systematic review and meta-analysis on the effectiveness of Baduanjin exercise for knee OA, results through WOMAC showed improvements in pain, stiffness and physical function [40]. In this pilot study, Baduanjin may not directly improve one’s physical activity level, however, indirect improvements in different components of health-related quality of life (pain relief, emotional role, and mental health) do contribute much relief for degenerative and long-term joint disease in ageing populations. Patients with OA knee often have symptomatic painful knee that affect their function. One of the advantages of this Tai Chi exercise is that the movements focus on the upper limb and core muscles and places less stress to the affected knee. This method of exercise is different from traditional physiotherapy exercise that focus on strengthening lower limb. Knee pain improvement was achieved by multi-disciplinary education program and Tai Chi exercise as described in this pilot study. Tai Chi exercise alone will not be able to improve knee pain.

High percentages of patients’ satisfaction and recommendation to the program are important findings in this study. The compliance (~ 90% of participants completed the whole course) and adherence rates are high. Local community accept the fact that Tai Chi are fit for elderly and movements are gentle for physically compromising personnel as well. We admit that those individuals are at their advanced knee OA (KL grade 3-4) and these patients are probably experiencing high level of pain. Our patients understand that a promised level of physical exercise is far better than being bed-bound. Tai Chi program signifies that Tai Chi does not cause any significant detriment to knee OA patients, and participating in any level of physical activity intervention that may show promise for symptom management.

We invited all participants to complete CSQ-8 at the end of the Tai Chi class (i.e., one time point), but not after the education program, or months after the multi-disciplinary education program. We aim at collecting satisfaction percentages after the program at this stage. Multiple point satisfaction outcome comparisons would be introduced in further studies.

Further studies are recommended to prove the effectiveness of a simple and reliable Tai Chi exercise (Baduanjin) together with a multidisciplinary education program for patients with knee OA. First, the true effect can only be validated by introducing a control or comparison group. Second, apart from Baduanjin, other versions of Tai Chi, for example, Yijinjing, and Wuqinxi can be introduced to compare the effects from other versions of Tai Chi and explore extra benefits from a modification and combination of the two need to be tested (comparison groups).

### Limitations of this study

This was a single-centre single-arm pre-post test design study which limited the data generalizability. This is a pilot study describing a multi-disciplinary condition management programme combined with Tai Chi. Four-week education program plus 8-week Tai Chi class are relatively short periods, especially if patients are encouraged to continue performing exercises daily over several months. Compliance to exercise over time is always an important issue influencing the program outcomes. Further study would be warranted through a randomised controlled trial including comparative and control groups to investigate the effectiveness of the multi-disciplinary education program. IPAQ short form, instead of the full version, was used to ease participants’ burden. This, however, generates insufficient responsiveness to reflect accurate physical activity changes. Response bias was also one of the limiting factors [41]. Patient satisfaction evaluation needs to carry out before and after an intervention.

## Conclusions

This is a pilot study to evaluate the proposed community-based, multi-disciplinary education program and a Tai Chi class on a small number of knee OA patients. The education program is a 1-month 2-h health talks and demonstrations conducted by different medical and health professionals. A 2-month (8-week) Tai Chi program (Baduanjin) which was an 8 Section Brocade Tai Chi formed the exercise component. After 6 months of completing the 8-week program, pain and emotional role were significantly improved. Physical ability and mental health tended to improve with time. Over 85% of our knee OA patients are very or highly satisfied with the multi-disciplinary education program. They feel that the program provides much information on self-management and improve their health outcomes towards their painful joints. The regimen of education program accompanying a Tai Chi exercise is proven feasible with high patients’ satisfaction. Introducing a control or comparison group would be the next approach. Different versions of Tai Chi exercises can be the option to follow studies.

## Data Availability

The datasets used and/or analysed during the current study are available from the corresponding author upon request.

## Abbreviations

Baduanjin: 8 Section Brocade Tai Chi
OA: Osteoarthritis
TKA: Total Knee Arthroplasty
EULAR: The European League against Rheumatism
KL: Kellgren and Lawrence Grading Scale
MET: Metabolic Equivalent of Task
IPAQ: International Physical Activity Questionnaire
SF-36v2: The Optum Short Form Quality of Life Questionnaire
WOMAC: The Western Ontario and McMaster Universities Arthritis Index
CSQ-8: The Client Satisfactory Questionnaire Scales
